# Comparable immune escape capacity for NB.1 with that of JN.1 variant and survey of infection with severe acute respiratory syndrome coronavirus 2 variants among Chinese *Felis silvestris catus*

**DOI:** 10.3389/fimmu.2026.1766267

**Published:** 2026-01-27

**Authors:** Youhua Yuan, Yiman Geng, Qiyuan Zhu, Bingfu Sun, Junhong Xu, Xiaohuan Mao, Xiaohuan Zhang, Wenqian Tian, Jing Zhao, Peiming Zheng, Lan Gao

**Affiliations:** 1 Department of Special Laboratory, Henan Provincial People’s Hospital, People’s Hospital of Zhengzhou University, and People’s Hospital of Henan University, Zhengzhou, Henan, China; 2 Department of PCR, Henan Provincial People’s Hospital, People’s Hospital of Zhengzhou University, and People’s Hospital of Henan University, Zhengzhou, Henan, China; 3 Department of Laboratory, Huaxian People’s Hospital, Anyang, Henan, China; 4 Department of Diagnosis and Treatment, Zhengzhou Municipal Fuchong Pet Hospital, Zhengzhou, Henan, China; 5 Department of Laboratory, Henan Provincial People’s Hospital, People’s Hospital of Zhengzhou University, and People’s Hospital of Henan University, Zhengzhou, Henan, China

**Keywords:** enzyme-linked immunosorbent assay, *Felis silvestris catus*, immune escape, JN.1, NB.1, pseudovirus neutralisation test

## Abstract

**Background:**

Neutralising antibodies and infection with the newest severe acute respiratory syndrome coronavirus 2 (SARS-CoV-2) variant NB.1 in Chinese *Felis silvestris catus* remains unclear. This study compared the capability of neutralising antibodies in serum against the NB.1 variant prevalent in 2025 with that of the JN.1 variant circulating in 2024 among ill Chinese *Felis silvestris catus*, and determined whether they could be infected with SARS-CoV-2 variants.

**Methods:**

A total of 392 serum samples from ill cats were subjected to enzyme-linked immunosorbent assay (ELISA) to detect the concentration of total antibodies against the receptor-binding domain of SARS-CoV-2; 40 serum samples screened positive by ELISA were subjected to pseudovirus neutralisation test to detect the titres of neutralising antibodies against the JN.1 and NB.1 variants, and 132 throat swab samples from ill cats were screened using specific reverse transcription polymerase chain reaction.

**Results:**

The geometric mean neutralising titres against the total, NB.1, and JN.1 Omicron variants were 9.51 (95% confidence interval: 7.34–12.3), 24.26 (18.84–31.23), and 48.79 (36.51–65.21) among 40 serum samples from ill cats, respectively. Therefore, neutralisation assays against JN.1 and NB.1 indicated 5.1- and 2.6-fold reductions in neutralising antibody titres, respectively, compared with the total antibody. Additionally, NB.1 showed a 2.91-fold reduction in neutralising antibody titres compared with JN.1. None of the throat swabs from the 132 ill cats were found to be infected with SARS-CoV-2 variants.

**Conclusions:**

NB.1 showed increased immune escape capacity in serum compared with JN.1 among Chinese *Felis silvestris catus*, suggesting that researchers should include the NB.1 antigen in COVID-19 vaccine candidates.

## 1 Introduction

Since the emergence of severe acute respiratory syndrome coronavirus 2 (SARS-CoV-2) in 2019, it has continually mutated and produced several notable variants of concern, including Omicron and its subvariants (1, 2) (e.g., BA.2, BA.5, XBB, EG.5, JN.1, XFG and NB.1). XFG and NB.1, currently the predominant lineages circulating in Europe, America, and Asia as of November 2025, are characterised by an extremely high transmission rate and significant immune evasion but relatively reduced pathogenicity (3). According to data from the Chinese Center for Disease Control and Prevention and our previous study (4), the SARS-CoV-2 variants prevalent in China in 2024 were JN.1 and its subvariant JN.1.18.2. However, since April 2025, the prevalent SARS-CoV-2 variant in China has mutated to the subvariant NB.1.8.1 of NB.1, accounting for >95% of all coronavirus disease 2019 (COVID-19) cases. Based on our previous research, the immune escape ability of the JN.1 variant in Chinese individuals who received booster vaccinations decreased by more than tenfold compared with that of the prototype strain. Although it is known that the immune escape ability of the KP.2 variant is similar to that of JN.1 (4), no reports exist on the immune escape ability of the NB.1 variant in humans or animals.

Since 2020, studies have reported that wild animals such as white-tailed deer and minks have tested positive for antibodies against SARS-CoV-2 (5, 6), whereas few reports describe such infection in domestic pet cats and dogs (7–10). In China, there are no reports describing neutralising antibodies against the newest SARS-CoV-2 variant NB.1, although a survey has examined SARS-CoV-2 variants in domestic pets such as cats. Therefore, with an aim to provide a reference for the origin and evolution of SARS-CoV-2, this study was conducted to determine whether neutralising antibodies against JN.1, prevalent in 2024, and NB.1, circulating in 2025, were present among serum samples from ill *Felis silvestris catus* in China. The infection status of the animals was determined using collected throat swab samples, followed by specific reverse transcription polymerase chain reaction (RT-PCR).

## 2 Materials and methods

### 2.1 Samples and flow of study

**Figure 1:**
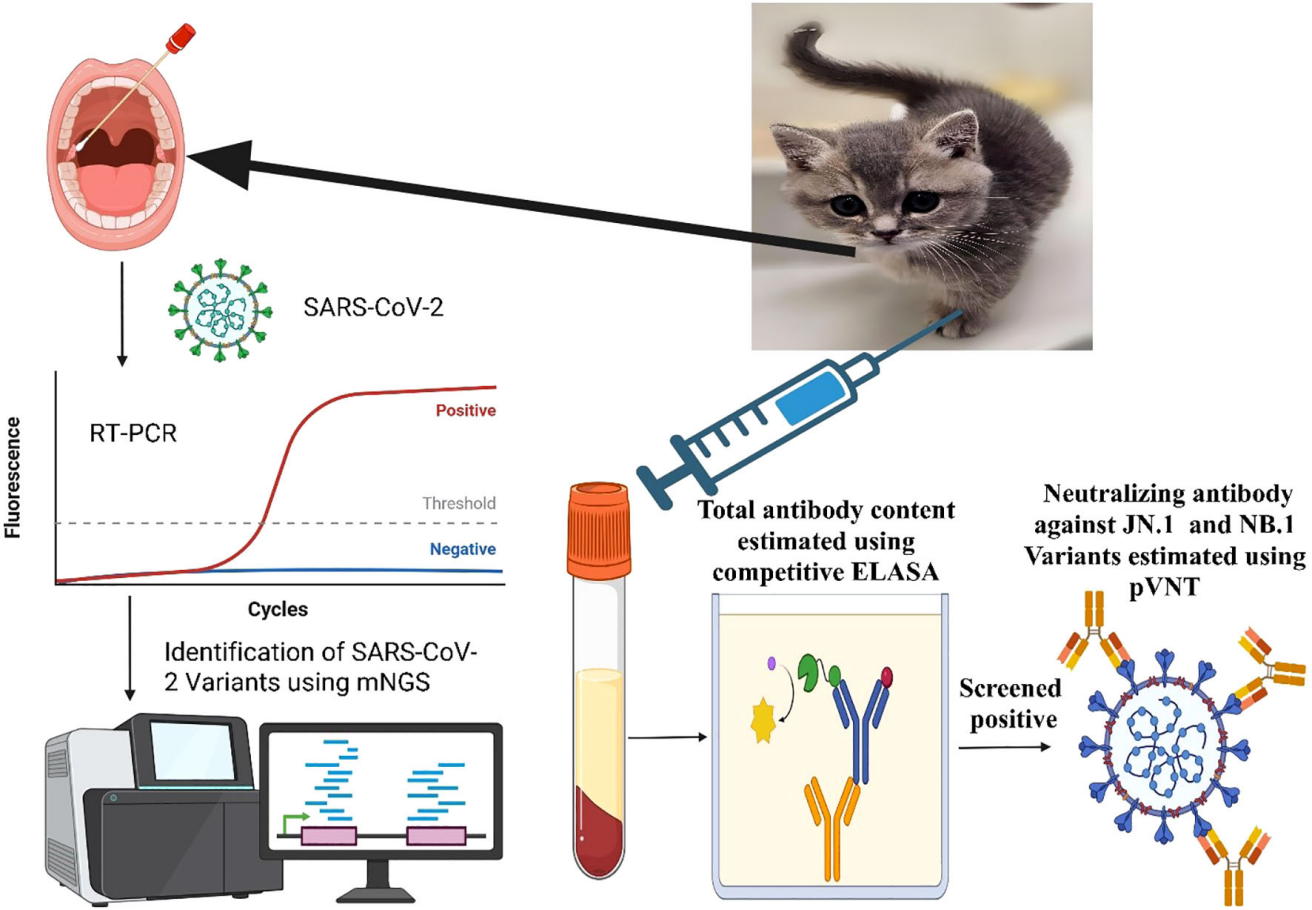
Flow chart of this study.

Between 2 August 2024, and 16 May 2025, 392 serum samples were collected from ill domestic cats in the Fuchong Pet Hospital in Zhengzhou City, central China (**Supplemntary File 1**, **Supplementary Figure S1**). To verify whether ill *Felis silvestris catus* were infected with SARS-CoV-2 using RT-PCR, an additional 132 throat swab samples were collected from the same pet hospital between August and December 2024. If positive samples were found to be infected with SARS-CoV-2 by RT-PCR, they were sent to the Beijing Macro and Micro Test Companies to further validate the SARS-CoV-2 variants through metagenomic next-generation sequencing (mNGS). Enzyme-linked immunosorbent assay (ELISA) was used to detect the total concentration of neutralising antibodies against the receptor-binding domain (RBD) of SARS-CoV-2 in June 2025 (11). After screening using ELISA, 40 positive cat samples were subjected to simultaneous pseudovirus-neutralising antibody testing against the JN.1 and NB.1 variants in October 2025. Data on the positivity rate of SARS-CoV-2 infection among inpatients and outpatients from August 2024 to May 2025 were obtained from the Ruimei Electronic Laboratory Information System of Henan Provincial People’s Hospital (Shanghai Ruimei Software Company, Shanghai, China). Further details regarding the study flow are presented in **Figure 1**.

### 2.2 Serum pseudovirus neutralisation test and ELISA

Pseudotyped viruses were generated by transfecting 293T cells with spike protein expression plasmids (JN.1 and NB.1 variants) and infecting them with G*ΔG-VSV (4, 8). Serum neutralisation titres were assessed via pVNTs. The *S* gene mutations and pseudovirus strains used are provided in **Supplementary File 1**, **Supplementary Table S1**. Serum samples were initially diluted at 1:30 or 1:10, followed by three-fold serial dilutions up to 1:7, 290. The 50% neutralisation dilution (ND50) was determined using the Reed–Muench method, with a limit of detection (LOD) of 1:10. Titres below the LOD were assigned a value of half the LOD, and an ND50 >1:30 was considered positive.

Total neutralising antibodies were measured using a commercial ELISA kit (anti-SARS-CoV-2 S kit; Shanghai GeneoDx Biotechnology Co., Ltd., Shanghai, China), according to the manufacturer’s protocol (10, 12). Absorbance was read using a universal microplate reader (DNM-9602; Beijing Pulong Co., Ltd., Beijing, China). A concentration >6.5 IU/mL was set as the positive threshold, and all values >100 IU/mL were reported as 100 IU/mL.

### 2.3 Screening positive samples infected with SARS-CoV-2 from cat throat samples and identification of variants

Throat swab samples from domestic cats were tested for the presence of SARS-CoV-2 using RT-PCR (Shanghai Zhijiang Biotechnology Co., Ltd.), with a cycle threshold (Ct) value ≤44 defined as positive. The screened positive samples were further subjected to mNGS to identify SARS-CoV-2 variants at the Beijing Macro and Micro Test Company (Beijing, China).

### 2.4 Ethics

The study protocol was reviewed and approved by the Institutional Review Board/Ethics Committee of Henan Provincial (approval number: 20210051; date: 24 May 2021). The studies were conducted in accordance with the local legislation and institutional requirements. Written informed consent was obtained from the owners for the participation of their animals in this study. As no human specimens were used and no drug interventions were performed on patients, written informed consent from patients was waived.

### 2.5 Statistics

Summary statistics for geometric means with 95% confidence intervals (CIs) are presented. Statistical significance between paired groups and subgroups was assessed using the Friedman H test and adjusted by the Bonferroni method for continuous variables. Additionally, Pearson’s χ^²^ or Fisher’s exact test was used for categorical variables. The kappa value obtained from the paired Pearson’s χ^²^ test was used to characterise the consistency between ELISA and pVNT for detecting neutralising antibodies against SARS-CoV-2 variants. A Kappa value >0.75 was regarded as indicating good consistency between the two methods. Spearman’s rank correlation and r-values were utilised to evaluate the correlation between two continuous variables. Hypothesis testing was two-sided, and statistical significance was set at *p* < 0.05. SPSS (version 25.0; IBM Corp., Armonk, NY, USA) and GraphPad Prism (version 8.0; La Jolla, CA, USA) were used for statistical analyses and plotting, respectively.

## 3 Results

### 3.1 Comparison of neutralising antibodies against total, JN.1, and NB.1 variants in sera from Chinese Felis silvestris catus

**Figure 2:**
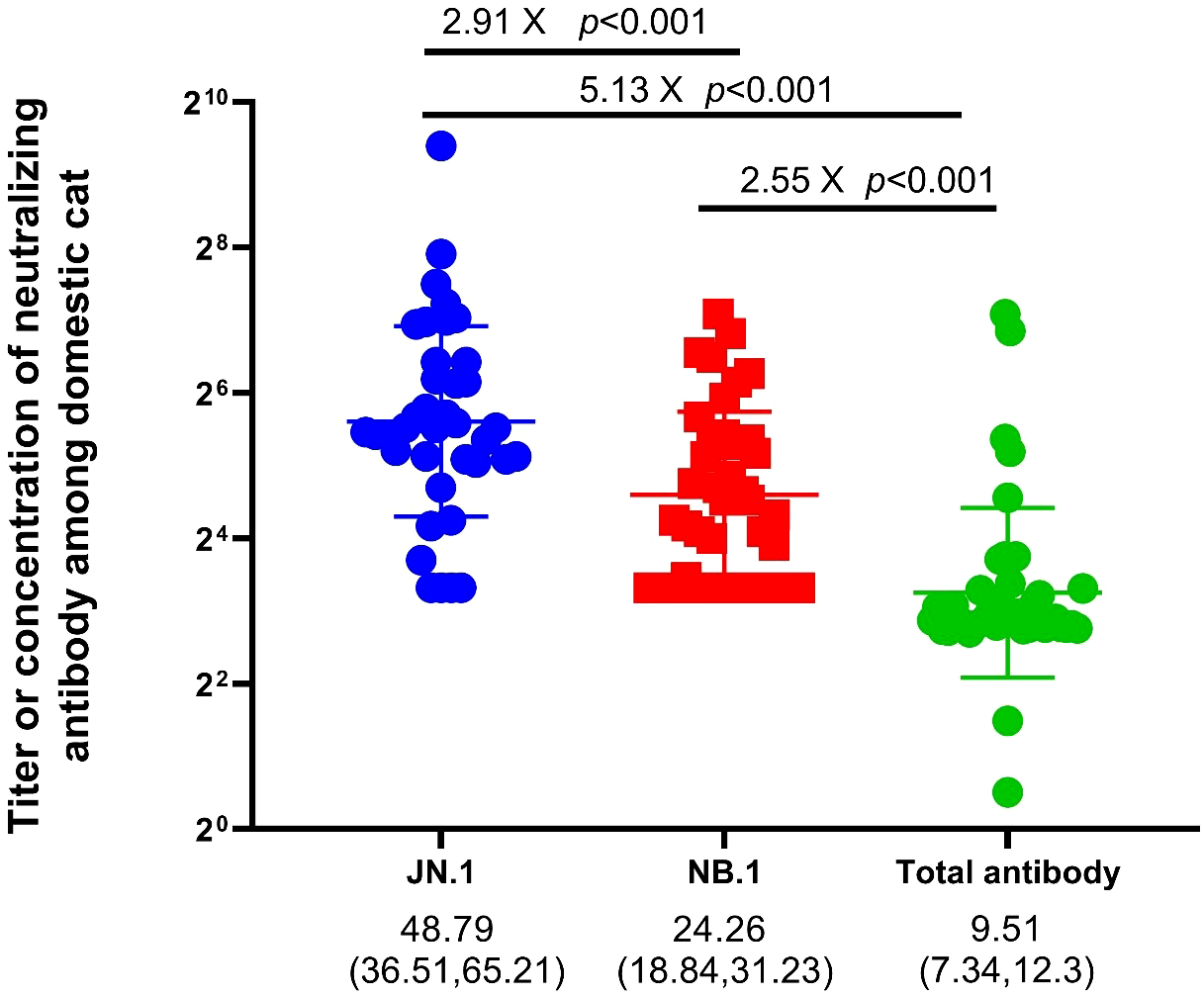
Comparison of neutralising antibody titres against JN.1, NB.1, and total IgG among Chinese Felis silvestris catus.

**Figure 3:**
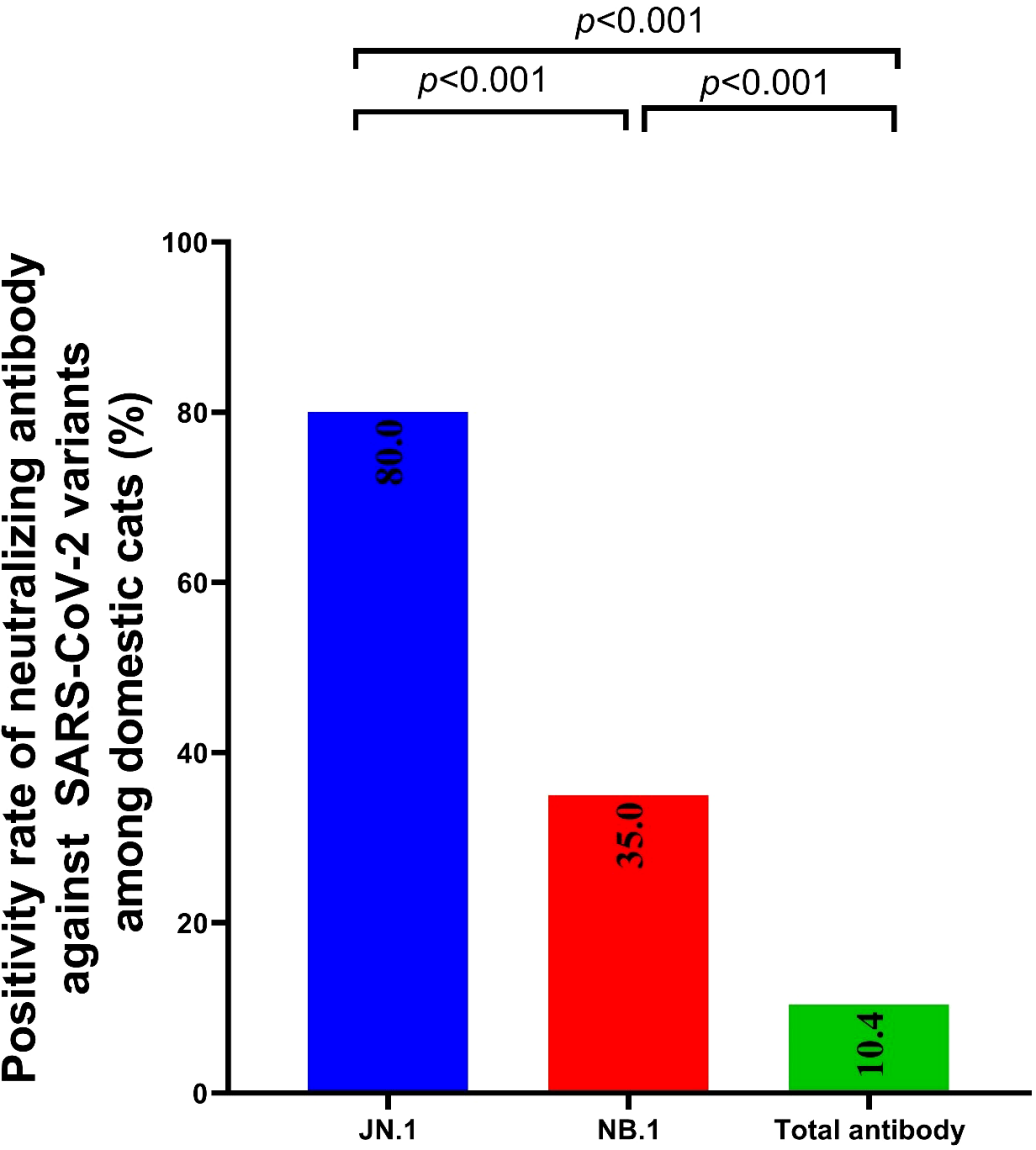
Comparison of positive neutralising antibody rate against JN.1, NB.1, and total IgG among Chinese Felis silvestris catus.

**Figure 4:**
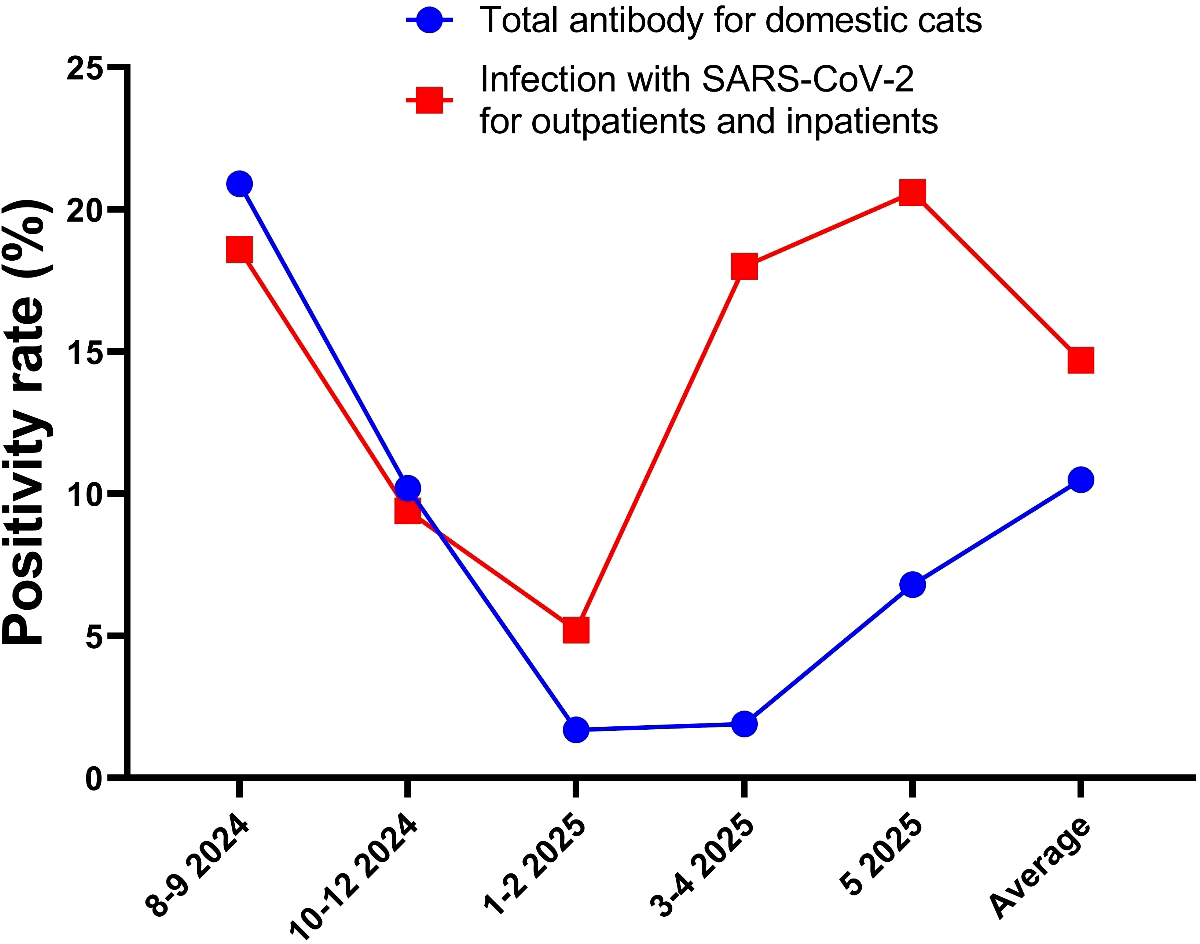
Comparison of positive total IgG of neutralising antibody rate among Chinese Felis silvestris catus with infection rate of SARS-CoV-2 among inpatients and outpatients from August 2024 to May 2025 in Henan Provincial People’s Hospital.

Sera from 392 domestic cats showed markedly reduced neutralisation against the Omicron variants JN.1 and NB.1 compared with total antibodies. The geometric mean neutralising titres were 9.51 (95% CI: 7.34–12.3) for total antibodies, 24.26 (18.84–31.23) for NB.1, and 48.79 (36.51–65.21) for JN.1 (**Figure 2**), corresponding to a 5.1-fold reduction for JN.1 and a 2.6-fold reduction for NB.1 relative to total antibodies. The NB.1 variant (2025) also showed a 2.91-fold lower titre than JN.1 (2024). Neutralising antibody titres and positivity rates differed significantly among the groups (adjusted *p* < 0.001), with positivity rates of 80% (JN.1), 35% (NB.1), and 10.4% (total antibodies) (**Figures 2**, **3**). These results confirmed that the immune escape capacity of NB.1 was weaker than that of JN.1. Moreover, the trend in the increase in total antibody positivity in cats aligned with the rise in SARS-CoV-2 infection rate in patients from August 2024 (18.6%) to May 2025 (20.6%) (*p* < 0.001; **Figure 4**; **Supplementary File 1**, **Supplementary Tables S2**, **S3**).

### 3.2 Comparison of consistency between ELISA and pVNT for detecting neutralising antibodies against SARS-CoV-2 variants in cat serum

**Figure 5:**
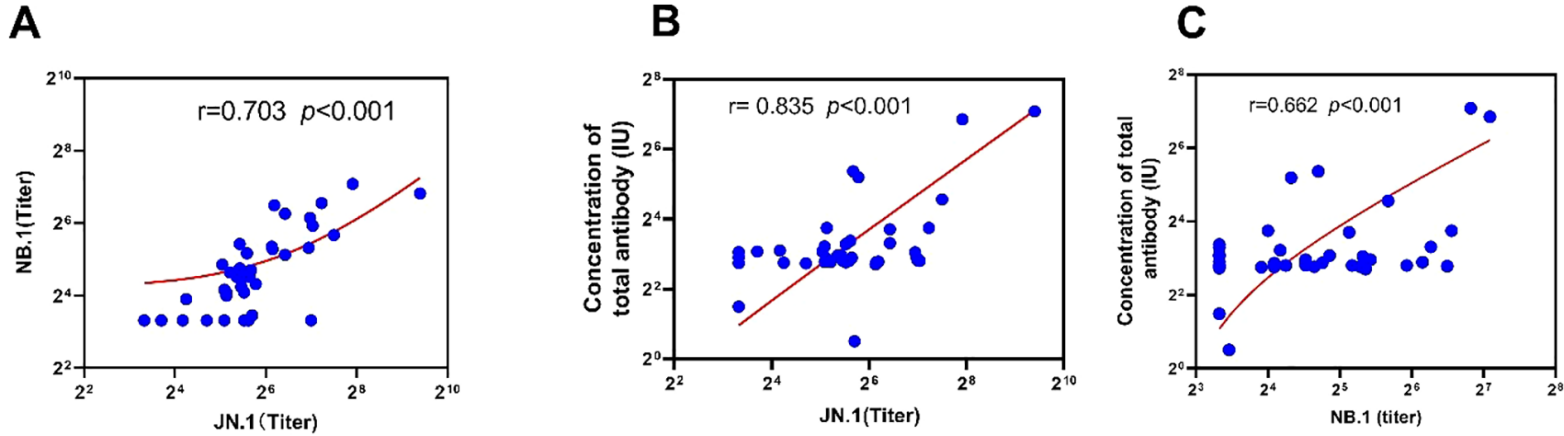
Relationship between the titre of serum neutralising antibody or concentration among JN.1, NB.1, and total IgG antibody from Chinese Felis silvestris catus. **(a)** between JN.1 and NB.1; **(b)** JN.1 and total antibody; **(c)** NB.1 and total antibody.

We compared the consistency between ELISA and pVNT for detecting neutralising antibodies against JN.1 and NB.1 variants in 40 serum samples from domestic cats. The Kappa value between ELISA and JN.1 was 0.13, which was higher than that between ELISA and NB.1, which was 0.055; however, both Kappa values were less than 0.75, indicating that the two methods did not show good consistency for detecting neutralising antibodies in serum from domestic cats (**Figure 3**; **Supplementary File 1**, **Supplementary Tables S4, S5**). A high positive correlation was observed among neutralising antibodies against the total, JN.1, and NB.1 variants (r = 0.703, r = 0.835, and r = 0.662, *p* < 0.001, respectively) (**Figures 5a–c**).

### 3.3 Status of SARS-CoV-2 infection in throat samples from Chinese domestic cats

None of the 134 throat samples from ill domestic cats collected from August 2024 to December 2024 tested positive for SARS-CoV-2.

## 4 Discussion

This study was conducted to assess the neutralising activity of domestic cat serum (central China, Aug 2024–May 2025) against Omicron JN.1 and NB.1 and determine whether these variants evaded immunity from initial SARS-CoV-2 infection. To our knowledge, this is the first study to report the neutralisation capacity post-JN.1 infection and against emerging NB.1 in Chinese domestic cats. Although all 132 cat throat swabs (Aug–Dec 2024) tested negative for SARS-CoV-2, possibly due to limited sample size and duration, this still constitutes the first relevant survey in this population.

Our results showed that the positivity rate of neutralising antibodies against JN.1 was the highest at 80% (32/40), followed by NB.1 at 35% (14/40), and total antibodies at 10.4% (41/392) among the two detected variants and total antibodies in Chinese domestic cats. Even though the positivity rate of antibodies from cats differed from that in other countries, such as Thailand, Japan, Turkey, Portugal, and the USA (13–17), this trend coincided with the prevalent characteristics of SARS-CoV-2 variants from 2024 to 2025 in the Chinese population. Data from the Chinese Centre for Disease Control and Prevention and our previous study indicated that JN.1 and its sub-lineage JN.1.18.2 were the first circulating variants in 2024, accounting for 48.25% in China (https://ngdc.cncb.ac.cn/ncov/monitoring/country/China) and 52.9% in Henan province (3), central China. However, since January 2025, NB.1 and its sub-lineage NB.1.8.1 have replaced JN.1 and become the most prevalent variant in China, accounting for 97.5% in October 2025 (https://www.chinacdc.cn/jksj/xgbdyq/202511/t20251105313287.html).

Our data showed that the neutralising antibody titre against the NB.1 variant in domestic cats was 2.9 times lower than that against the JN.1 variant, and 5.1 times lower than the total antibody titre. This finding indicates that the immune escape ability of the NB.1 variant is higher than that of JN.1, suggesting that SARS-CoV-2 has been continuously evolving since its emergence in 2019 (18). Consequently, cats and humans previously infected with the JN.1 variant in 2024 may be susceptible to reinfection by the newly prevalent NB.1 variant and its sub-lineage NB.1.8.1 in 2025 (3). This hypothesis is supported by data on the variants monitored by the Chinese Centre for Disease Control and Prevention and our detection data from August 2024 to May 2025 (19) (**Figure 5**; **Supplementary File 1**, **Supplementary Tables S2**, **S3**).

Our present results show that the inconsistency between the use of ELISA and pVNT is similar to that observed in our previous study (4); this is mainly related to differences between the principles of the technologies and the targeted SARS-CoV-2 variants. ELISA uses a competitive principle to detect neutralising antibodies in serum against the RBD of SARS-CoV-2, which is equivalent to detecting the unmutated prototype strain, whereas pVNT detects neutralising antibodies in serum against SARS-CoV-2 variants. Based on these experimental principles, the pVNT is considered the gold standard for determining whether a person or animal has been infected with a specific SARS-CoV-2 variant or for assessing the neutralising capacity of serum against a particular variant. In contrast, ELISA can only preliminarily indicate SARS-CoV-2 infection in a person or animal and cannot identify the infecting variant. As ELISA is less expensive than pVNT (20), we first used ELISA to perform preliminary screening of the 392 cat serum samples and then conducted pVNT against the JN.1 and NB.1 variants on the 40 specimens that tested positive. The aim was to investigate whether the newly emerging NB.1 variant in 2025 would have a stronger immune evasion capability (21). The results confirmed that the NB.1 variant increased immune evasion capability, suggesting that in the future, researchers should either include the NB.1 antigen in COVID-19 vaccine candidates or design a broad-spectrum COVID-19 vaccine to address the increasingly emerging SARS-CoV-2 variants (22, 23).

This study has certain limitations. First, only a small number of cat throat swab specimens were obtained to screen for SARS-CoV-2 infection (132 samples), and the study duration was short, lasting only 4 months. Furthermore, this period may not have coincided with the peak of the COVID-19 pandemic. Therefore, no cat samples tested positive for SARS-CoV-2. These results indicate that cats in close contact with humans may be less susceptible to SARS-CoV-2 than other animals, such as minks (3). However, this hypothesis requires a larger sample size for further verification. Second, we only collected samples from cats. As dogs, pigs, cows, and wild rats may also be susceptible to SARS-CoV-2 (5, 8), we plan to collect blood, throat swabs, and other tissues from these domestic animals and wild animals in future studies to detect neutralising antibodies against different SARS-CoV-2 variants and determine whether the animals are infected with SARS-CoV-2. Third, we did not collect clinical symptom data from the sick cats. Therefore, we were unable to compare the differences in clinical symptoms among cats positive for neutralising antibodies against the JN.1 and NB.1 variants. In future studies, we will ensure the collection of clinical symptom data and analyse the differences among cats positive for different neutralising antibody variants.

## 5 Conclusions

We found that cats, similar to humans, can have a certain proportion of samples positive for neutralising antibodies against the prevalent strains JN.1 and NB.1. Furthermore, the neutralising antibody capacity of cat serum against the NB.1 variant circulating in 2025 was significantly lower than that against the JN.1 variant prevalent in 2024. Additionally, as no SARS-CoV-2-positive samples were detected in the limited number of cat throat swab specimens, the hypothesis that pet cats may act as reservoirs or sources of transmission should be further investigated using larger sample sizes and broader geographic areas. Our results provide a new perspective on the evolution of SARS-CoV-2 in humans and animals, and on the origin of the virus.

## Data Availability

The original contributions presented in the study are included in the article/**Supplementary Material**. Further inquiries can be directed to the corresponding authors.
