# Supplementary material for: Comparable immune escape capacity for NB.1 with that of JN.1 variant and survey of infection with severe acute respiratory syndrome coronavirus 2 variants among Chinese Felis silvestris catus
Source: Front Immunol. 2026 Jan 27;17:1766267. doi: 10.3389/fimmu.2026.1766267 (PMC12886400; doi:10.3389/fimmu.2026.1766267)
Supplement: Supplementary file 1 [file Table1.docx]

Supplementary Material

1. Supplementary Figures
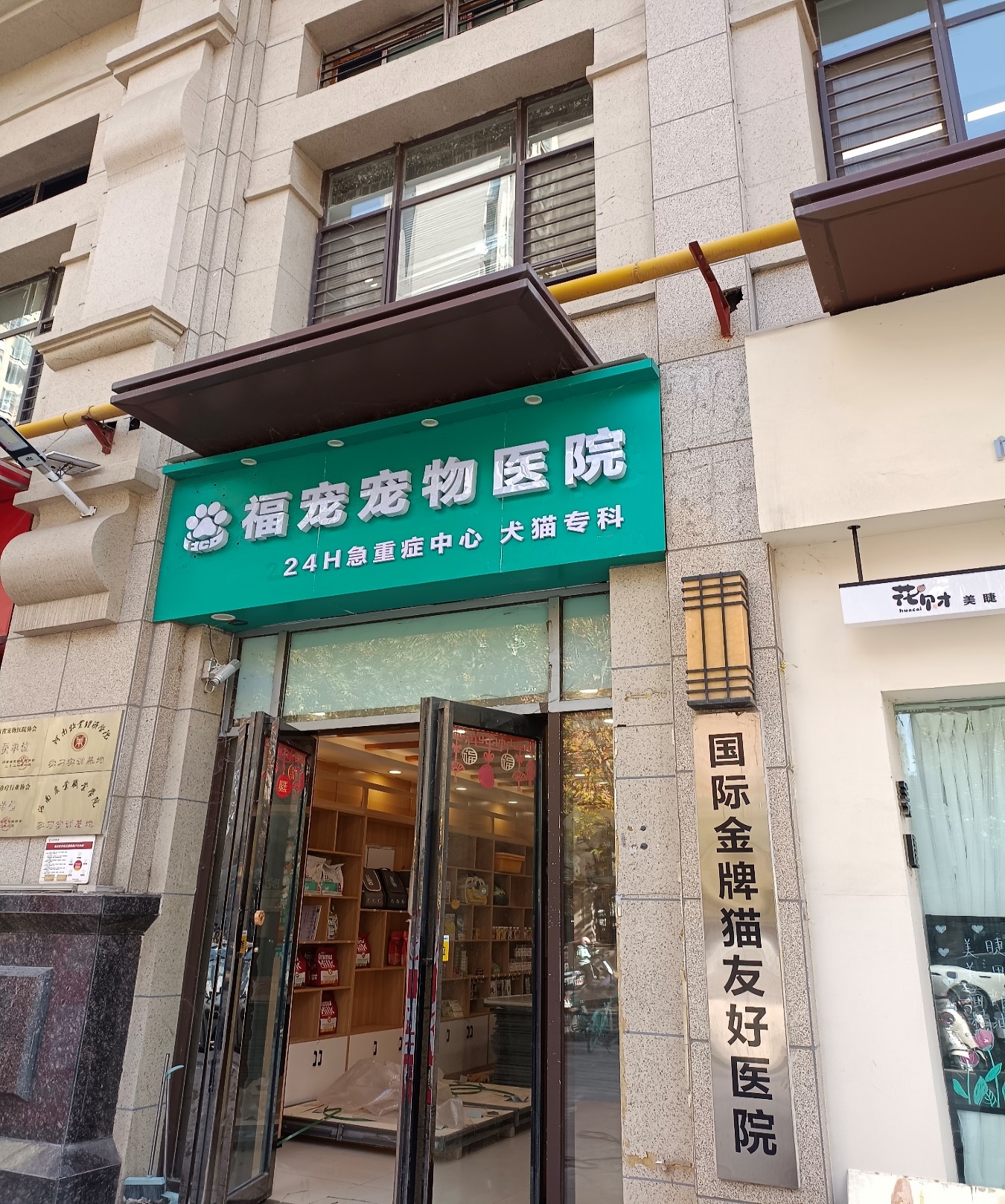


**Supplementary Figure S1.** Zhengzhou Municipal Fuchong Pet Hospital.

# 2. Supplementary Tables

Table S1. Pseudotyped viruses used for neutralisation titre detection and mutation sites of the *S* gene

| **Risk Classification by WHO** | **Pangolin**  **lineage** | **Pseudotyped**  **virus** | **Mutation site of *S* gene (based on GenBank sequence MN908947)** |
| --- | --- | --- | --- |
| VOI | BA.2.86 | JN.1 | T19I R21T L24S del25/27 S50L del69/70 V127F G142D del144/144 F157S R158G N211I del212/212 V213G L216F H245N A264D I332V G339H K356T S371F S373P S375F T376A R403K D405N R408S K417N N440K V445H G446S N450D L452W L455S N460K S477N T478K N481K del483/483 E484K F486P Q498R N501Y Y505H E554K A570V D614G P621S H655Y N679K P681R N764K D796Y S939F Q954H N969K P1143L |
| VOM | BN.1.3.1 | NB.1 | T19I R21T L24S del25/27S50L F59S del69/70V127F G142D del144/144 F157S R158G G184S N211Idel212/212 V213G L216F H245N A264D I332V G339H R346T K356T S371F S373P S375F T376A R403K D405N R408S K417N N440K V445H G446S N450D L452W L455S F456L N460K S477N T478I N481Kdel483/483 E484K F486P Q498R N501Y Y505H E554K A570V D614G P621S H655Y N679K P681R N764K D796Y S939F Q954H N969K V1104L P1143L |

Table S2. Positivity rate of SARS-CoV-2 infection in inpatients and outpatients at Henan Provincial People’s Hospital

| Date | Aug to Sep 2024 | Oct to Dec 2024 | Jan to Feb 2025 | Mar to Apr 2025 | May 2025 | Total |
| --- | --- | --- | --- | --- | --- | --- |
| Total number  of people tested | 408 | 543 | 599 | 635 | 845 | 3030 |
| Number of SARS  CoV-2-positive individuals | 76 | 51 | 31 | 114 | 174 | 446 |
| Positivity rate of  SARS-CoV-2 infection (%) | 18.6 | 9.4 | 5.2 | 18.0 | 20.6 | 14.7 |

Table S3. Positivity rate of total antibodies against SARS-CoV-2 among domestic cats in Central China

| Date | Aug to Sep 2024 | Oct to Dec 2024 | Jan to Feb 2025 | Mar to Apr 2025 | May 2025 | Total |
| --- | --- | --- | --- | --- | --- | --- |
| Total number of domestic cats tested | 110 | 127 | 58 | 53 | 44 | 392 |
| Number of antibodies against the RBD of SARS-CoV-2-positive cats | 23 | 13 | 1 | 1 | 3 | 41 |
| Positivity rate of antibody against RBD of SARS-CoV-2 infection (%) | 20.9 | 10.2 | 1.7 | 1.9 | 6.8 | 10.5 |
| Number of antibodies against JN.1-positive cats | 19 | 10 | 0 | 1 | 3 | 33 |
| Positivity rate of antibody against JN.1 infection (%) | 82.6 | 76.9 | 0 | 100 | 100 | 80.0 |
| Number of antibodies against NB.1-positive cats | 6 | 5 | 1 | 0 | 2 | 14 |
| Positivity rate of antibody against NB.1 infection (%) | 26.1 | 38.5 | 100 | 0 | 66.7 | 35.0 |

Table S4. Comparison of results between ELISA and pVNT for detecting neutralising antibodies against SARS-CoV-2 JN.1

|  | ELISA | | |  |
| --- | --- | --- | --- | --- |
|  |  | + | - | Total |
| pVNT against JN.1 | + | 31 | 1 | 32 |
|  | - | 7 | 1 | 8 |
|  | Total | 38 | 2 | 40 |
| Statistic result | Kappa = 0.13, *p* = 0.364 | | | |

ELISA, enzyme-linked immunosorbent assay; pVNT, pseudovirus neutralisation test

Table S5. Comparison of results between ELISA and pVNT for detecting neutralising antibodies against SARS-CoV-2 NB.1

|  | ELISA | | |  |
| --- | --- | --- | --- | --- |
|  |  | + | - | Total |
| pVNT against NB.1 | + | 14 | 0 | 14 |
|  | - | 24 | 2 | 26 |
|  | Total | 38 | 2 | 40 |
| Statistic result | Kappa = 0.055, *p*=0.533 | | | |

ELISA, enzyme-linked immunosorbent assay; pVNT, pseudovirus neutralisation test
